# Small RNA-based plant protection against diseases

**DOI:** 10.3389/fpls.2022.951097

**Published:** 2022-08-18

**Authors:** Özlem Bilir, Deniz Göl, Yiguo Hong, John M. McDowell, Mahmut Tör

**Affiliations:** ^1^Department of Biotechnology, Trakya Agricultural Research Institute, Edirne, Turkey; ^2^Department of Biology, School of Science and the Environment, University of Worcester, Worcester, United Kingdom; ^3^Research Centre for Plant RNA Signaling, College of Life and Environmental Sciences, Hangzhou Normal University, Hangzhou, China; ^4^School of Plant and Environmental Sciences, Virginia Tech, Blacksburg, VA, United States

**Keywords:** HIGS, SIGS, sRNA, plant protection, pathogens

## Abstract

Plant diseases cause significant decreases in yield and quality of crops and consequently pose a very substantial threat to food security. In the continuous search for environmentally friendly crop protection, exploitation of RNA interferance machinery is showing promising results. It is well established that small RNAs (sRNAs) including microRNA (miRNA) and small interfering RNA (siRNA) are involved in the regulation of gene expression *via* both transcriptional and post-transcriptional RNA silencing. sRNAs from host plants can enter into pathogen cells during invasion and silence pathogen genes. This process has been exploited through Host-Induced Gene Silencing (HIGS), in which plant transgenes that produce sRNAs are engineered to silence pest and pathogen genes. Similarly, exogenously applied sRNAs can enter pest and pathogen cells, either directly or *via* the hosts, and silence target genes. This process has been exploited in Spray-Induced Gene Silencing (SIGS). Here, we focus on the role of sRNAs and review how they have recently been used against various plant pathogens through HIGS or SIGS-based methods and discuss advantages and drawbacks of these approaches.

## Introduction

All plant species are routinely challenged by pests and pathogenic microorganisms including viruses, bacteria, oomycetes, fungi, parasitic plants, or nematodes (Jones and Dangl, 2006;Bilir et al., 2019). Many plant diseases cause substantial damage to crop production, reducing crop quality and leading to substantial economic losses worldwide (Zhu et al., 2019). Concurrently, human population growth has created increasing demand for safe, nutritious and accessible foods produced in an environmentally sustainable manner. Eco-friendly plant protection strategies are an integral component of sustainable intensification to obtain increased crop yields (Liu et al., 2020).

One such strategy is to exploit plant RNA interference machinery (RNAi), which is an evolutionarily conserved regulatory mechanism to combat pathogens and control expression of endogenous genes (Hameed et al., 2017; Iqbal et al., 2020; Liu et al., 2020).

In this review, we focus on two different approaches to exploit RNAi for disease control: Host-Induced Gene Silencing (HIGS) and Spray Induced Gene Silencing (SIGS). HIGS is a transgenic technology, where double-stranded RNA (dsRNA) of a pathogen pathogen gene is expressed in the plant and the derivative small RNAs are taken up by the interacting pathogen, triggering silencing of the targeted gene (Sang and Kim, 2020). In contrast, SIGS relies on external application (e.g., spraying) of dsRNAs or small RNAs (sRNAs) that target pathogen genes (Bilir et al., 2019). Both approaches show promise for disease control. Here, we highlight recent advances and challenges.

## Small RNA

Small RNAs have been recognized as key genetic and epigenetic regulators functioning in processes ranging from the modification of DNA methylation to the modulation of the abundance of coding or non-coding RNAs in various organisms (Qin et al., 2017). Regulatory sRNAs are involved in diverse biological programs, processes, and pathways in response to developmental signals, pathogen infection and pest attacks (Chen et al., 2014). In plants, sRNAs are versatile regulators of development, growth and response to biotic and abiotic stresses (Yang and Huang, 2014) and are comprised of two major classes – microRNAs (miRNAs) and small interfering RNAs (siRNAs). miRNAs are derived from endogenous MIR genes that are transcribed by RNA polymerase II into primary miRNAs (pri-miRNA) having partially double-stranded (ds) stem–loop structures. Processed, mature miRNAs are 20–22 nucleotides in length. siRNAs are 21–24 nucleotides in length and are processed from long double-stranded RNA (Guleria et al., 2011). In contrast to miRNAs, siRNAs can be be produced from endogenous genes and exogenous sources such as viruses, transposons and transgenes (Guleria et al., 2011). Many sub-classes of siRNAs have been described in plants, including tasiRNAs, phasiRNAs, natsiRNAs, and hc−siRNAs (for detailed information seeHuang et al., 2016).

## RNAi

RNA interference comprises potent, evolutionarily conserved genetic regulatory mechanisms to silence gene expression (Baulcombe, 2004). One of the major features of RNAi is the production of sRNAs of 21–30 nt in length that can regulate gene expression in a sequence-specific manner. siRNAs generated from dsRNA can guide transcriptional and post-transcriptional gene silencing (TGS and PTGS, respectively;De Felippes, 2019). The most extensively studied sRNA regulators control PTGS by base pairing with mRNA targets, thereby causing their degradation, inhibiting translation, or both (Vaucheret et al., 2001; Chen et al., 2014). Contrastingly, TGS is based on DNA methylation and histone modifications that form a heterochromatic environment around the target gene, restricting access to transcription factors and RNA polymerase (Vaucheret et al., 2001).

## Plant immunity

Plants have a complex defense system against various invading pathogens and pests. Plant defense mechanisms against microbes is activated by receptor proteins that recognize two broad categories of pathogen-derived signals. One category, termed microbe-associated molecular patterns (MAMPs) is comprised of many classes of molecules, typically recognized by cell surface receptors, activating MAMP-trggered immunity (MTI). The second category is comprised of secreted pathogen virulence proteins called effectors, typically recognized inside plant cells, to activate effector-triggered immunity (ETI) (Jones and Dangl, 2006; Tör et al., 2009). Plant sRNAs have been demonstrated as critical regulators in the reprogramming of gene expression during both PTI and ETI downstream of recognition (Katiyar-Agarwal et al., 2007; Uddin et al., 2017). Moreover, pathogen effector proteins have been shown to target plant RNAi machinery as a means of disabling immunity (Xiong et al., 2014). Finally, recent studies demonstrated that sRNAs can themselves act as effectors by trans-kingdom RNAi (ckRNAi) by translocating from pathogen to host, wherein they silence host genes (Dunker et al., 2020). Altogether, sRNAs play pivotal roles in the link between hosts and their interacting pathogens (Koch et al., 2020). As such, sRNAs have received considerable attention as tools to enhance immunity and attenuate pathogen virulence.

## Host-induced gene silencing

**FIGURE 1:**
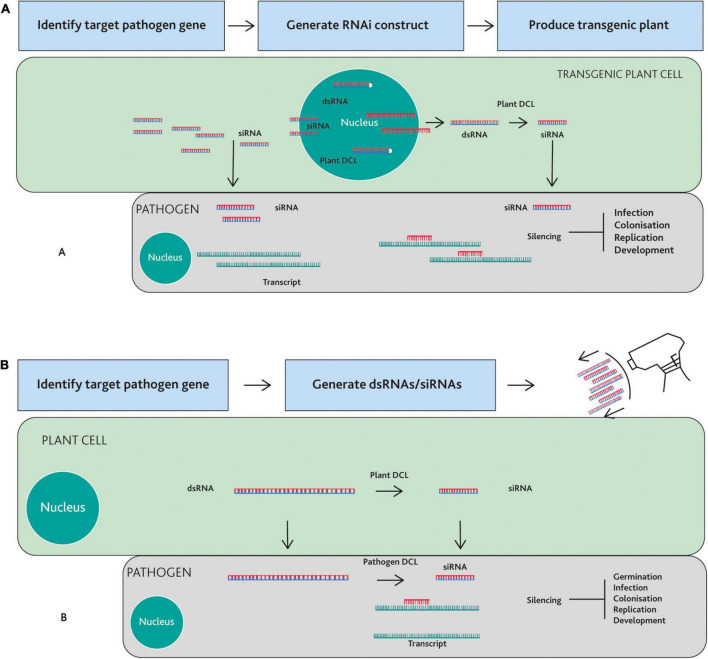
Strategies to control plant pathogens using Host-Induced Gene Silencing (HIGS) or Spray Induced Gene Silencing (SIGS). **(A)** HIGS: A target gene is identified in the pathogen and an RNAi construct is generated from the part of the target gene sequence. Plants are transformed with the construct and transgenic lines are selected. The transgenic plant cell produces double stranded RNA (dsRNA), which are subjected to the cleavage by the plant Dicer-like (DCL) proteins within the nucleus and/or cytoplasm, producing small interfering RNA (siRNAs) that translocate to the pathogen cell. siRNAs guide the RNA silencing machinery of the pathogen cell to silence mRNAs from the target gene. **(B)** SIGS: dsRNAs or siRNAs targeting a pathogen gene are synthesized and sprayed onto plants. Sprayed dsRNAs are directly taken up by fungal cells or are taken up by plant cells and then transferred into fungal cells. Plant or pathogen Dicer-like (DCL) proteins cleaves these dsRNAs into siRNAs. In this case, dsRNAs are likely cleaved in the cytoplasm although this process could also occur in the nucleus. Resultant siRNAs guide the RNA silencing machinery of the pathogen cell to silence targeted mRNA. Depending on the function of the target gene, HIGS- or SIGS-mediated gene silencing can lead to inhibition of spore germination, infection, colonization, replication, or development of the pathogen.

Host-induced gene silencing is a plant transgene-mediated technique, in which plants express an RNAi construct designed against a specific gene(s) endogenous to the pathogen (Santala and Valkonen, 2018). The transgenes typically produce dsRNA or a hairpin-structured dsRNA construct, with sequence identify to a specific pathogen gene. The transgenes can be stably transformed into the host plant; Alternatively, systems for transient transformation can be employed. The transgenic plant transcribes dsRNAs that are processed into siRNAs, which in turn are translocated into the plant pathogens (Figure 1A; Sang and Kim, 2020).

The crucial step for a successful HIGS strategy is the identification of suitable target genes in the pathogen (Koch and Kogel, 2014). This is often achieved by analyzing transcriptomic data or a genome database. Once the target genes are identified, an *in vitro* assay on pathogen cultures could be performed using artificially synthesized siRNAs/dsRNAs that solely target the gene under study. Subsequently, an expression vector is constructed, which, when expressed in plants, generates the dsRNA and siRNA population precisely targeting pathogen transcripts (Ghag, 2017). The other critical step in HIGS is to ensure that the dsRNA and corresponding siRNA species do not exert off-target effects (Koch and Kogel, 2014).

In principle, HIGS could provide a robust tool to down-regulate the expression of key fungal pathogen genes that are required for disease progression in the host (Majumdar et al., 2017). Moreover, HIGS might be used to control multiple diseases of a given crop because constructs can be designed that contain multiple (“stacked”) RNAi transgenes designed against different pathogens (Nowara et al., 2010). Another convenience is that the genes could be designed as “race-specific” or broad-spectrum based on the degree of sequence conservation within the targeted region. For example, Guo et al. (2019) used HIGS to target the *MoAp1* in *Magnaporthe oryzae* (*M. oryzae*) and achieved improved resistance to 11 different strains of the pathogen with a single construct. Finally, such genes should not affect other plant traits as long a sufficient care is taken to avoid off-target effects. As such, this approach can provide a valuable complement to conventional breeding, or chemical controls, thereby supporting the goal of environmentally friendly and durable resistance in crops (Ghag, 2017; Zhu X. et al., 2017).

## Spray-induced gene silencing

Although convenient in many contexts, transgenes are not necessary for ectopic activation of gene silencing in pathogens or pests. Highly effective gene silencing can also be achieved by exogenous application of sRNAs (Ghosh et al., 2018). When the exogenous dsRNAs are applied to plants, they move into pathogen cells and induce RNAi. The RNAs sprayed on the plant surfaces have at least two possible pathways to get into fungal or oomycete cells (Figure 1B; Koch et al., 2016; Wang et al., 2016). Once entering plant cells, dsRNAs are cleaved into sRNAs by plant Dicer-like proteins (DCLs). At the same time, some of these dsRNAs and sRNAs in the plant cells are also transferred into the cells of the fungal or oomycete pathogen. After this trans-kingdom spread, dsRNAs are processed into sRNAs mainly by the fungal DCLs. Alternatively, externally applied dsRNAs and sRNAs can be taken up directly by fungal or oomycete cells. Again, dsRNAs may be cleaved into sRNAs by fungal or oomycete DCLs (Figure 1B).

In practice, exogenous sRNA application has been carried out *via* surface treatments, such as spraying (Dalakouras et al., 2016) or soaking (San Miguel and Scott, 2016), and invasive methods, such as infiltration (Numata et al., 2014), injection, soil/root drench, or petiole absorption (Ghosh et al., 2017; Dalakouras et al., 2018). Foliar sprays, trunk injections, root drench, or delivery from clay granules can also be used for sustained release of dsRNA (Ghosh et al., 2018). Another promising approach, exemplified in some of the studies described below, is to use transgenic bacteria as a source of sRNA (Niño-Sánchez et al., 2021).

SIGS is an efficient, highly flexible, innovative strategy for crop protection against pathogen infection (Koch et al., 2019;Hu et al., 2020;Sang and Kim, 2020). The main difference and advantage relative to HIGS is that transgenic plants are not necessary, thereby saving the time and money of developing transgenic plants and avoiding the onerous, expensive deregulation as well as issues with acceptance of transgenic plant. However, there are still gaps in our knowledge of the mechanisms underlying SIGS and hurdles to implementation certainly exist (see discussion below andWerner et al., 2020).

## sRNA-based antiviral defense

Plants recognize virus infection by an antiviral defense system called virus-induced gene silencing (VIGS,Santala and Valkonen, 2018). Manipulation of this process has become a weapon of choice against viruses in plants (Arif et al., 2012). For example, transgenes producing RNA that triggers RNAi have been at the forefront of resistance to viruses since the mid-1980s. One of the most successful applications of this technology are papaya in Hawaii with transgenes against the otherwise intractable papaya ringspot viruses (Gonsalves, 2006). Table 1 compiles a short list of the latest successful examples.

**TABLE 1:** Summary of HIGS and SIGS applications for control of viral pathogens.

Type of gene silencing	Pathogen	Host	RNA Type	Target Gene(s)	Main Effect(s)	References
HIGS	Wheat streak mosaic virus (WSMV)	Wheat	hpRNA	*NIa gene*	Immunity to WSMV infection	Fahim et al., 2010
	Wheat streak mosaic virus (WSMV)	Wheat	amiRNA-1 amiRNA-2 amiRNA-3 amiRNA-4 amiRNA-5	*5′ UTR region*, *ORF pipo region of P3 cistron*, *P1 gene*, *P3 cistron*, *HCpro gene*	Immunity to WSMV infection	Fahim et al., 2012
	Rice tungro bacilliform virus (RTBV)	Rice	dsRNA	*ORF IV*	Decreased accumulation of RTBV in rice plants	Tyagi et al., 2008
	Rice dwarf virus (RDV)	Rice	dsRNA	*Pns12* *Psn4*	Resistance to RDV	Shimizu et al., 2009
	Rice stripe virus (RSV)	Rice	hpRNA	*CP* *SP* *CP/SP*	Enhanced resistance against RSV	Ma et al., 2011
	Rice stripe virus (RSV)	Rice	hpRNA	*pC2* *pC3* *pC4* *p4*	Immunity to infection for *pC2* and *pC3*, non-resistance for *pC4* and *p4*	Shimizu et al., 2011
	Potato virus X (PVX) Potato virus Y (PVY) Potato leaf roll virus (PLRV)	Potato	hpRNA	*ORF2 (PVX)* *HC-Pro (PVY)* *CP (PLRV)*	Broad-spectrum resistance to all three viruses	Arif et al., 2012
	Potato virus X (PVX) Potato virus Y (PVY) Potato virus S (PVS)	Potato	hpRNA	*CP*	Nearly 100% resistance against PVX, PVY, and PVS infection	Hameed et al., 2017
	African cassava mosaic virus (ACMV)	Tobacco	dsRNA	*AC1* *AC2* *AC4* *BC1*	Resistance to bipartite geminivirus infection	Patil et al., 2016
	Soybean mosaic virus (SMV) Bean yellow mosaic virus (BYMV)	Tobacco	hpRNA	*CP*	Increased resistance of soybean to SMV and BYMV	Thu et al., 2016
	Ugandan cassava brown streak virus (UCBSV) and Cassava brown streak virus (CBSV)	Tobacco	amiRNA	*P1 (CBSV and UCBSV)* *P3 (CBSV and UCBSV)* *CI (UCBSV)* *Nib (CBSV and UCBSV)*	Resistance against UCBSV and CBSV	Wagaba et al., 2016a
	Ugandan cassava brown streak virus (UCBSV) and Cassava brown streak virus (CBSV)	Tobacco	amiRNA	*CP*	High levels of resistance to both viruses	Wagaba et al., 2016b
	Tomato leaf curl Gujarat virus (ToLCGV)	Tobacco	siRNAs	*COP9* *PPRP* *GPX-1* *USP* *HSTF-B4* *ARF18* *WRKY-6* *SDR*	Decreased expression of subunit-7 of CSN complex and *WRKY6*, increased expression of USPA-like protein	Prakash et al., 2020
	Cucumber necrosis virus (CNV)	Tobacco	dsRNA	*CP*	Interfered with chloroplast-mediated plant defense	Alam et al., 2021
	Pepper mottle virus (PepMoV)	Tobacco	dsRNA	*HC-Pro* *NIb*	Defended the host against viral infection instantly and inhibited viral growth effect	Yoon et al., 2021
	Cowpea severe mosaic virus (CPSMV), Cowpea aphid-borne mosaic virus (CABMV)	Cowpea	hpRNA	*32K protein* *CP*	Resistance to CPSMV and CABMV	Cruz and Aragao, 2014
	Soybean mosaic virus (SMV)	Soybean	hpRNA	*HC-Pro*	Strong viral resistance	Kim et al., 2016
	Cassava brown streak virus (CBSV), Uganda cassava brown streak virus (UCBSV)	Cassava	hpRNA	*CP*	High levels of resistance to both viruses	Beyene et al., 2017
Co-inoculation-spraying	Sugarcane Mosaic Virus (SCMV)	Sugarcane	dsRNA with bacterial expression	*CP*	Inhibited SCMV infection	Gan et al., 2010
SIGS (spraying)	Pea seed borne mosaic virus (PSbMV)	Pea	dsRNA	*CP*	Significant short-term reduction in the virus concentration, reduced viral titer	Safarova et al., 2014
Bioclay- spraying	Pepper mild mottle virus (PMMoV) and cucumber mosaic virus (CMV)	Tobacco Cowpea	dsRNA with bacterial expression	*RP gene of PMMoV, 2b of CMV2b*	Increased stability and protection period	Mitter et al., 2017
Bioclay-spraying	Potyvirus bean common mosaic virus (BCMV)	Tobacco Cowpea	dsRNAs with bacterial expression	*NIb* *CP*	Protected plants from aphid-transmission of BCMV	Worrall et al., 2019

hpRNA, hairpin RNA; amiRNA, artificial microRNA; dsRNA, double-stranded RNA; siRNA, small interfering RNA.

Spray-induced gene silencing has been explored for protection of plants against viruses. Some studies have investigated the use of crude extracts of bacterially expressed dsRNA to protect plants against virus infections. Tenllado et al. (2003) tested a foliar spray of crude extracts of bacterially expressed dsRNA for protection of *N. benthamiana* against Pepper mild mottle virus (PMMoV) or Plum pox virus (PPV). Following viral inoculation at 5 days after dsRNA application, the dsRNAs caused specific degradation of viral RNA and protection of the plants against viral infection. Notably, spraying plants with a crude bacterial preparation resulted in the dsRNA providing similar protection. In a similar experiment, Gan et al. (2010) sprayed maize with a crude extract of bacterially synthesized dsRNA derived from two fragments of the Sugarcane Mosaic Virus (SCMV) CP gene. Plants were inoculated with the virus 3 days after spraying with a half-strength extract and inhibition of viral infection was observed. In a different approach, Mitter et al. (2017) constructed a topical spray of layered double hydroxide (LDH) clay nanosheets loaded with dsRNA (BioClay) for protection of tobacco and cowpea against cucumber mosaic virus (CMV) or pepper mild mottle virus (PMMoV). In contrast to naked dsRNA, which can be unstable, a single spray of bacterially expressed dsRNA loaded on BioClay could provide virus protection for at least 20 days, presumably due to sustained release of the dsRNA (Table 1).

## sRNA-based protection of plants from bacterial invasion

Although bacteria do not have an RNAi machinery similar to eukaryotes, they do contain gene regulation mechanisms that employ RNA molecules. These include CRISPR RNAs that inhibit the uptake of foreign DNA and small RNA, which bind to proteins or base pair with target RNAs (Waters and Storz, 2009). Escobar et al. (2001) developed a HIGS-based strategy to improve resistance to *Agrobacterium* crown gall based on knowledge of the *iaaM* and *ipt* oncogenes, which are required for tumor formation. *Arabidopsis thaliana* and *Lycopersicon esculentum* plants were transformed with constructs to initiate RNAi against these two oncogenes. Following infection with *A. tumefaciens*, the transformed *A. thaliana* and *L. esculentum* plants displayed 0.0–1.5% tumorigenesis and 0.0 – 24.2% tumorigenesis, respectively, compared to nearly 100% in controls. Subsequent molecular investigation confirmed significant reduction in the accumulation of both *iaaM* and *ipt* transcripts in the transgenic line as compared with the wild type (Escobar et al., 2001). Similar work has also been carried out with walnut, and the silencing *iaaM* and *ipt* genes has been shown to decrease crown gall formation (Walawage et al., 2013).

Another approach to control bacterial pathogens using HIGS is the targeting of the host susceptibility genes. For example, the rice gene *Os8N3* provides susceptibility to *Xanthomonas oryzae* pv. *oryzae* strain PXO99*^A^* (Yang et al., 2006). When *Os8N3* was targeted with a HIGS system, transgenic plants became resistant to this strain of X. oryzae pv. *oryzae*.

Mahmoudi and Soleimani (2019) used HIGS and revealed that the expression of the *aiiA* gene in potato could reduce the *N*-acylhomoserine lactone quorum sensing signal and consequently enhance resistance to bacterial soft rot disease (Table 2). To our knowledge, there is no SIGS-based studies against bacterial plant pathogens.

**TABLE 2:** Summary of HIGS applications for control of bacterial pathogens.

Pathogen	Host	RNA type	Target gene(s)	Main effect(s)	References
*A. tumefaciens*	Arabidopsis Tobaco	siRNA	*iaaM* *ags*	Inhibited infection	Dunoyer et al., 2006
*A. tumefaciens*	Rice	hpRNA	*OsPDS* *OsSLR1*	Triggered degradation of target transcripts in the adjacent tissues	Andrieu et al., 2012
*A. tumefaciens*	Walnut	dsRNA	*iaaM* *ipt*	Decreased crown gall formation	Walawage et al., 2013
*A. tumefaciens*	Plum Apricot	hpRNA	*iaaM* *ipt*	Induces resistance to crown gall disease in plum but not in apricot	Alburquerque et al., 2017
*Pectobacterium carotovorum* (Soft rot)	Potato	miRNA	*aiiA*	Induced resistance to the early stage of bacterial pathogenesis	Mahmoudi and Soleimani, 2019

hpRNA, hairpin RNA; dsRNA-Double-stranded RNA; siRNA, Small interfering RNA; miRNA, micro RNA.

## Host-induced gene silencing-based protection of plants from fungal infection

Small RNAs strategies have been tested against plant pathogenic fungi and, to a lesser extent, oomycetes (Table 3). A large number of studies have focused on fungal pathogens of wheat and barley, using HIGS from transient viral expression vectors or from stably integrated transgenes. For example, Panwar et al. (2013) evaluated the efficiency of HIGS induced by transient transformation of wheat with a barley stripe mosaic virus (BSMV) construct targeting a mitogen-activated protein kinase (MAPK), a cyclophilin, and a calcineurin regulatory subunit in the wheat leaf rust fungus *Puccinia triticina (Pt)*. They reported a reduction in endogenous transcript levels, suggesting the translocation of siRNA molecules from host to fungal cells. In addition, the subsequent disease suppression implicated the targeted fungal genes in pathogenicity. In another study from the same group, HIGS-based targeting of *PtMAPK1* or *PtCYC1* impaired fungal development and reduced disease severity in wheat (Panwar et al., 2018). Qi et al. (2017) used BSMV−mediated HIGS to silence the *PsCPK1* gene of *Puccinia striiformis* f. sp. *tritici* (*Pst*). They demonstrated that PsCPK1 is an important pathogenicity factor for *Pst*, and knockdown of *PsCPK1* led to decreased virulence of *Pst*. Similarly, Zhu X. et al. (2017) demonstrated *PsFUZ7* was a pathogenicity factor that regulated infection and development of *Pst*. They observed strong restriction of hyphal development and necrosis of plant cells (a possible defense response) within siRNA-producing host tissue. These examples demonstrate that transient transformation can be used to induce HIGS and test the functional importance of candidate fungal virulence genes. This approach can be used to quickly test candidate target genes for HIGS-based disease control before investing effort into creation of stable transgenic plants.

**TABLE 3:** Summary of HIGS and SIGS applications for control of fungal and oomycete pathogens.

Type of gene silencing	Pathogen	Host	RNA type	Target gene(s)	Main effect(s)	References
HIGS	*Puccinia triticina*/Wheat Leaf Rust	Wheat	siRNA	*PtMAPK1 PtCYC1 PtCNB*	Reduction in endogenous transcript levels	Panwar et al., 2013
	*Fusarium graminearum*/Fusarium head blight and Fusarium seedling blight	Wheat	hpRNA	*Chs3b*	Durable resistance to *Fusarium* head blight and seedling blight in wheat	Cheng et al., 2015
	*Puccinia striiformis f. sp. tritici*/Wheat Stripe Rust	Wheat	hpRNA	*PsCPK1*	Decreased virulence of *Pst*	Qi et al., 2017
	*Puccinia striiformis* f. sp. *tritici*/Wheat Stripe Rust	Wheat	dsRNA	*PsFUZ7*	Strong restriction of hyphal development	Zhu X. et al., 2017
	*Fusarium graminearum*/Fusarium Head Blight	Wheat	miRNA	*FGSG_03101*	Suppressed invasion of *F. graminearum*	Jiao and Peng, 2018
	*Puccinia triticina*/Wheat Leaf Rust	Wheat	hpRNA	*PtMAPK1* *PtCYC1*	Impaired fungal development and a reduction in disease severity and progression in wheat plants	Panwar et al., 2018
	*Blumeria graminis f. sp. tritici*/Wheat Powdery Mildew	Wheat	dsRNA	β*2-tubulin*, *SvrPm3a1/f1* *Bgt_Bcg-6 Bgt_Bcg-7*	Impaired virulence of *B. g. tritici* at the haustorial stage	Schaefer et al., 2020
	*Puccinia striiformis* f. sp. *tritici*/Wheat Stripe Rust	Wheat		*TaCSN5*	Broad−spectrum resistance to wheat stripe rust	Bai et al., 2021
	*Blumeria graminis*/Powdery Mildew	Wheat Barley	mRNA	*Avra10*	Reduction in haustorium formation	Nowara et al., 2010
	*Blumeria graminis* f. sp. *hordei*/Barley Powdery Mildew	Barley	siRNA	*CSEP0062* *CSEP0081* *CSEP0145* *CSEP0216* *CSEP0222* *CSEP0254* *CSEP0398*	Reduced rate of fungal penetration and haustoria formation	Ahmed et al., 2016
	*Blumeria graminis f. sp. hordei*/Barley Powdery Mildew	Barley	hpRNA	*CSEP0139 CSEP0182*	Reduced haustorial formation and size of the lesions	Li et al., 2021
	*Rhizoctonia solani/*Sheath Blight	Rice	hpRNA	*RPMK1-1* *RPMK1-2*	Reduced *R. solani* infection	Tiwari et al., 2017
	*Magnaporthe* oryzae/Rice Blast Fungus	Rice	siRNA	*MoABC1* *MoMAC1* *MoPMK1*	Inhibited disease development and reduced the transcription of the genes	Zhu L. et al., 2017
	*Magnaporthe oryzae*/Rice Blast Fungu*s*	Rice	asiR1245 asiR1362 asiR1115	*MoAP1*	Inhibited disease development and reduced the transcription of targeted fungal genes	Guo et al., 2019
	*Aspergillus flavus*/Aspergillus Ear Rot	Maize	siRNAs	*aflR*	Reduction to aflatoxin accumulation in transgenic maize	Masanga et al., 2015
	*Aspergillus flavus*, *Aspergillus parasiticus*	Maize	siRNAs	*aflC*	Reduced aflatoxin below the regulatory threshold	Thakare et al., 2017
	*Aspergillus flavus/*Aspergillus Ear Rot	Maize	siRNA	*aflM*	Reduced aflatoxin contamination	Raruang et al., 2020
	*Phytophthora infestans*/Potato Late Blight	Potato	hpRNA	*PiGPB1* *PiCESA2* *PiPEC* *PiGAPDH*	Enhanced resistance to the late blight disease	Jahan et al., 2015
	*Phytophthora infestans*/Potato Late Blight	Potato	siRNA	*Avr3a*	Inhibition of target gene expression in *P. infestans*	Sanju et al., 2015
	*Sclerotinia sclerotiorum*/White Mold	Tobacco	hpRNA	*Chs*	Reduction in the level of CHS endogenous transcripts	Andrade et al., 2016
	*F. oxysporum* f. sp. *cubense*/Fusarium Wilt	Banana	ihpRNA	*VEL* *FTF1*	Reduced growth and decreased pathogenesis	Ghag et al., 2014
	*Fusarium oxysporum*/Fusarium Wilt	Banana	ihpRNA	*Foc TR4 ERG6/11*	Inhibition of growth and development of *Foc TR4*	Dou et al., 2020
	*Fusarium oxysporum* f. sp. *lycopersici*/Tomato Fusarium Wilt	Tomato	hpRNA	*FOW2* *chsV*	Inhibited fungal growth and increased resistance to the pathogen	Bharti et al., 2017
	*Colletotrichum gloeosporioides*/Anthracnose	Chilly Tomato	siRNA	*CgCOM1*	Confers resistance against anthracnose disease in chilly and tomato	Mahto et al., 2020
	*Fusarium oxysporum f. sp. lycopersici*/Tomato Fusarium Wilt	Tomato	siRNA	*ODC*	Inhibited fungal growth and increased resistance to the pathogen	Singh et al., 2020
	*Verticillium dahliae*/Verticillium Wilt	Tomato Arabidopsis	hpRNA	*Ave1* *Sge1* *NLP1*	Reduced the disease	Song and Thomma, 2018
	*Fusarium graminearum*/Fusarium Head Blight	Arabidopsis and Barley	dsRNA	*CYP51*	Growth inhibition and alteration in fungal morphology	Koch et al., 2013
	*Fusarium oxysporum* f. sp. conglutinans/Anthracnose	Arabidopsis	dsRNA	*FRP1* *FOW2* *OPR*	Enhanced disease resistance	Hu et al., 2015
	*Fusarium graminearum/*Fusarium Head Blight	Arabidopsis	dsRNA	*FgCYP51A* *FgCYP51* *FgCYP51C*	Reduction in infection	Höfle et al., 2020
	*Fusarium graminearum*/Fusarium Head Blight	Arabidopsis	dsRNA	*CYP51A* *CYP51B* *CYP51C*	Inhibited growth of *F. graminearum*	Koch et al., 2020
	*Verticillium dahliae/Verticillium Wilt*	Tobacco Arabidopsis	sRNA	*VdAK*	Decreased mycelial growth and spore production during abiotic stress	Su et al., 2020
	*Phakopsora pachyrhizi*/Asian Soybean Rust	Soybean	siRNA	*ATC* *UN_1* *UN_2* *UN_3* *GCS_H* *PR_S16* *CRP_6* *PHR*	Reduced endogenous *P. pachyrhizi* transcript abundance, fungal biomass accumulation and the development of the disease symptoms; suppressed the expression of target genes	Hu et al., 2020
	*Fusarium oxysporum*, *Fusarium graminearum*	Soybean	ihpRNA	*CYP51B*	Mild or no symptoms in leaves and root of three lines of six transgenic lines and better plant development	Perez et al., 2021
	*Bremia lactucae*/Down Mildew Lettuce	Lettuce	siRNA	*HAM34* *CES1*	Reduced growth and inhibited sporulation of the pathogen	Govindarajulu et al., 2015
	*Rhizoctonia solani*/Brown Patch	Tall Fescue	dsRNA	*RNApoly* *Imbs* *Coh* *UbiE3*	Improved resistance against *R. solani* and reduced lesion size	Zhou et al., 2016
	*Fusarium graminearum*/Fusarium Head Blight	Purple False Brom	dsRNA	*Fg00677* *Fg08731* *CYP51*	Reduced pathogenicity of the fungus	He et al., 2019
	*Verticillium dahlia*/Verticillium Wilt	Cotton		*VdILV2* *VdILV6*	A dramatic reduction in pathogenicity	Wei et al., 2020
	*Rhizoctonia solani*/Sheath Blight	Tomato	dsRNA	*RS_CRZ1*	Significant reduction in disease symptoms and the depth of pathogen colonization	Ghosh et al., 2021
	*Cytospora chrysosperma*/Cytospora Canker	Canadian poplar		*Sge1*	Restricted vegetative growth, conidial reduction and lost pathogenicity	Han et al., 2021
	*Arbuscular mycorrhizal*	Medicago		*RiNLE1*	Suppression of defense−related gene expression and enhanced colonization levels	Wang et al., 2021
SIGS	*Fusarium graminearum*/Fusarium Head Blight	Barley	dsRNA	*CYP51A* *CYP51B* *CYP51C*	Inhibited the growth of the necrotrophic fungus	Koch et al., 2016
	*Fusarium graminearum*/Fusarium Head Blight	Barley	dsRNA	*CYP51A* *CYP51B* *CYP51C*	Reduced *F. graminearum* infection areas	Koch et al., 2020
	*Fusarium graminearum*/Fusarium Head Blight	Barley	dsRNA	*FgAGO1* *FgAGO2* *FgDCL1* *FgDCL2*	Reduced inhibition of fungal infection	Werner et al., 2020
	*Sclerotinia sclerotiorum*/White Mold	Barley	dsRNA	*Ss-ThioR* *Ss-TIM44* *Ss-CHC* *SS-AP2* *Ss-Arf72A* *Ss-FCHO1* *Ss-Amph* *Ss-VATPase* *Ss-eGFP*	Significantly reduced pathogen growth on plant	Wytinck et al., 2020
	*Hyaloperonospora arabidopsidis* (Hpa)/Downy Mildew	Arabidopsis	dsRNA	*Hpa-CesA*	Inhibited spore germination and infection	Bilir et al., 2019
	*Fusarium graminearum/*Fusarium Head Blight	Arabidopsis	dsRNA	*FgCYP51A* *FgCYP51* *FgCYP51C*	Increased disease resistance	Höfle et al., 2020
	*Botrytis cinerea*/Gray Mold Rot	Tomato Strawberry Grape Lettuce Onion Rose Arabidopsis	sRNAs dsRNA	*Bc-DCL1* *Bc-DLC2*	Inhibition of fungal growth, reduced symptoms of the disease and suppressed fungal DCL transcripts	Wang et al., 2016
	*Fusarium graminearum/*Fusarium Head Blight	Wheat	dsRNA	*Myosin 5*	Reduction in phenamacril-resistance	Song et al., 2018
	*F. asiaticum*, *B. cinerea*, *Magnaporthe oryzae*, *Colletotrichum truncatum*	Cucumber Soya Barley Wheat	dsRNA	*Fa*β*2Tub-3*	Decreased resistance to carbendazim fungicide, inhibited fungal growth and lead to crooked and multiple-branching mycelium	Gu et al., 2019
	*Botrytis cinerea*/Gray Mold Rot	Grapevine	dsRNA	*BcCYP51* *Bcchs1* *BcEF2*	Significant reduction in pathogen development	Nerva et al., 2020
	*Phakopsora pachyrhizi/*Soybean Rust	Soybean	dsRNA	*ATC* *GCS_H* *RP_S16*	Reduction in fungal biomass and a lower number of pustules on leaves	Hu et al., 2020
	*Sclerotinia sclerotiorum, Rhizoctonia solani, Aspergillus niger*, *Verticillium dahlia*, *Colletotrichum gloeosporioides*, *Trichoderma virens*, *Phytophthora infestans*	Lettuce Tomato Rose Grape	dsRNA	*VPS51* *DCTN1* *SAC1*	Inhibited the virulence of *B. cinerea, S. sclerotiorum*, *A. niger, R. solani*, reduced disease symptoms by *V. dahlia*, no significant reduction in targeted mRNA expression levels at the *P. infestans* inoculated, no inhibition of infection of *C. gloeosporioides*	Qiao et al., 2021
	*Phytophthora infestans/*Potato Late Blight	Potato	dsRNA	*SDH* *EF-1*α *GPI-HAM344* *PLD-3* *HSP-90*	Enhanced disease resistance and less sporulation	Sundaresha et al., 2021
	*Plasmopara viticola/*Grapevine downy mildew	Grapevine	dsRNA	*PvDCL1/2*	Reduced disease progress rate	Haile et al., 2021
	*Phytophthora infestans/*Potato late blight	Potato	dsRNA	*PiGPB1* *PiHmp1* *PiCut3* *PiEndo3*	Reduction in disease progression	Kalyandurg et al., 2021

hpRNA, hairpin RNA; ihpRNA, intron-containing hairpin RNA; miRNA, micro RNA; dsRNA, double-stranded RNA; siRNA, small interfering RNA; asiRNA artificial siRNA.

Schaefer et al. (2020) created stable transgenic wheat lines for HIGS against the powdery mildew pathogen *Blumeria graminis* f. sp. *tritici*. They targeted β*2-tubulin, SvrPm3a1/f1, Bgt_Bcg-6 and Bgt_Bcg-7* genes of *B.g. tritici*. Their results suggested that silencing of these three target effectors impaired virulence of *B.g. tritici* at the haustorial stage indicating each gene contributes to the pathogen’s virulence. Similarly, HIGS targeting the effector gene *Avra10* was used to protect wheat and barley against *Blumeria graminis* (Nowara et al., 2010). Ahmed et al. (2016) used HIGS in barley against 7 candidate secreted effector proteins of *B. graminis* f. sp. *hordei* (*Bgh*). They determined that only the silencing of *CSEP0081* and *CSEP0254* significantly reduced the rate of fungal penetration and haustoria formation, demonstrating their role as virulence factors (Table 2).

Jiao and Peng (2018) screened wheat sRNAs that could target the *F. graminearum* genome by silencing with BSMV. They found that a wheat microRNA (miR1023) could suppress the invasion of *F. graminearum* by silencing *FGSG_03101*, coding for an alpha/beta hydrolase in the fungus. Koch et al. (2013) evaluated the potential of HIGS targeting the fungal cytochrome *P450 lanosterol C-14*α*-demethylase* (*CYP51*) gene, which is important for ergosterol biosynthesis, to restrict infection of *Arabidopsis* and barley plants by *Fusarium* spp. They observed both growth inhibition and alteration in fungal morphology in axenic cultures of *F. graminearum* and restriction of mycelium formation on leaves expressing anti-CYP3 RNA. In another study, HIGS against cytochrome *P450 sterol 14*α*-demethylase* genes (*CYP51A* and *CYP51B*) in *Arabidopsis* inhibited the growth of *F. graminearum* (Koch et al., 2020). HIGS constructs targeting *F. graminearum* genes *Fg00677* and *Fg08731* in transgenic *Brachypodium distachyon* conferred resistance to this wheat pathogen, demonstrating that *Brachypodium* could be used as a facile test system for HIGS to enhance resistance in wheat (He et al., 2019).

Host-induced gene silencing also shows promise against *Fusarium* pathogens of dicot food crops. In banana, two *F. oxysporum* f. sp. *cubense* (*Foc*) gene were targeted using HIGS, resulting in decreased pathogenesis (Ghag et al., 2014). Similarly, Dou et al. (2020) reported inhibition of *Foc* growth and development on transgenic bananas with HIGS constructs that targeted two ergosterol biosynthetic genes. HIGS technology was also used against the tomato wilt pathogen *F. oxysporum* f. sp. *lycopersici*. In one study, *FOW2 and chsV* genes (Bharti et al., 2017), in another study, *ornithine decarboxylase* (*ODC*) were targeted (Singh et al., 2020); both studies reported that silencing these genes inhibited fungal growth and increased resistance to the pathogen.

Mahto et al. (2020) HIGS against the *Colletotrichum gloeosporioides COM1* (*CgCOM1*) gene, which is involved in fungal conidial and appressorium formation, inhibits the development of the appressorial/infection apparatus of the pathogenic fungi in chilly and tomato fruits and results in the prevention of manifestation of anthracnose disease in the plant.

Host-induced gene silencing also shows promise against the destructive rice blast disease caused by the fungus *Magnaporthe oryzae*. Zhu L. et al. (2017) targeted *MoABC1, MoMAC1*, and *MoPMK1* genes from *M. oryzae* to examine the effectiveness of transient, BMV-mediated HIGS in rice. They documented that mRNA abundance of the targeted genes was reduced and disease development was inhibited. In a SIGS-based approach against the same disease, Guo et al. (2019) showed silencing of *M. oryzae MoAP1* by feeding artificial siRNAs (asiRNAs) to *in vitro* cultures of *Magnaporthe oryzae*, which resulted in the inhibition of disease development and reduced the transcription of targeted fungal genes.

In an example of how multiple pathogen genes can be simultaneously targeted from a single HIGS transgene, Tiwari et al. (2017) transformed rice with a hairpin RNAi construct containing a fusion of two *Pathogenicity Map Kinase 1* (*PKM1*) genes, *RPMK1-1* and *RPMK1-2* of *Rhizoctonia solani*, which are essential for the formation of appressoria. The expression level of both *RPMK1-1* and *RPMK1-2* was significantly reduced in *R. solani* infecting transgenic rice lines. Zhou et al. (2016) transformed tall fescue with RNAi constructs of four “essential” genes from *R. solani*. They found that six of 19 transgenic plants showed improved resistance against *R. solani* and lesion size was decreased by as much as 90% indicating the value of multiple HIGS constructs for controlling the disease.

In a creative approach to reduce aflatoxin contamination of corn, Masanga et al. (2015) transformed plants with a hairpin construct targeting the aflatoxin biosynthesis transcription factor *aflR* of *Aspergillus flavus* and reported that *aflR* was downregulated and significantly lower levels of aflatoxins (14-fold) than those from wild type plants were observed. Thakare et al. (2017) determined that *aflC* gene encodes an enzyme in the *A. flavus* aflatoxin biosynthetic pathway and then transformed maize plants with an RNAi construct targeting the *aflC* gene. Following *A. flavus* infection, aflatoxin could not be detected in kernels from these RNAi transgenic maize plants, while non-transgenic control kernels showed high toxin loads. Recently, HIGS targeting *A. flavus aflM* gene encoding versicolorin dehydrogenase reduced aflatoxin contamination in transgenic maize under field conditions (Raruang et al., 2020). These results indicate that aflatoxin levels can be effectively reduced by targeting the mycotoxin biosynthetic pathway, without producing any overt off-target effects on the host crop plant.

The fungal pathogen *Verticillium dahliae* causes several destructive diseases. Hu et al. (2015) generated stable transgenic *Arabidopsis* plants expressing RNAi constructs targeting three *V. dahliae* virulence genes. *Verticillium* wilt disease was suppressed by two of the three targets in transgenic *Arabidopsis*. Targeting the same three genes in tomato with transient viral expression resulted in disease suppression from only one of the three genes. In cotton, HIGS against two *V. dahliae* acetolactate synthase genes reduced pathogenicity dramatically, indicating that these genes are essential for pathogenicity of *V. dahliae* (Wei et al., 2020). Song and Thomma (2018) investigated whether HIGS can be utilized to suppress *Verticillium* wilt disease by silencing three previously identified virulence genes of *V. dahliae* (*Ave1*, *Sge1*, and *NLP1*) through the host plants tomato and *Arabidopsis*. In these hosts, *V. dahliae* infection studies showed reduced *Verticillium* wilt disease in HIGS experiments targeting two of the three genes. Su et al. (2020) generated transgenic *N. benthamiana* and *A. thaliana* harboring *dsVdAK* to silence *VdAK* gene of *V. dahlia* by HIGS. They reported that *VdAK* was crucial for energy metabolism and that silencing of this gene decreased mycelial growth and spore production during abiotic stress. This study illustrates the important point that the effectiveness of sRNA-based protection can be affected positively or negatively by environmental conditions.

## Spray-induced gene silencing against fungal pathogens

In a pioneering study to test SIGS in multiple host plants against multiple fungal pathogens, Wang et al. (2016) applied Bc-sRNAs and dsRNAs onto the surface of fruits, vegetables and flowers; they determined that *B. cinerea* could take up both sRNAs and dsRNAs directly, and both could induce silencing of *B. cinerea DCL1* and *DCL2* genes. They found significant inhibition of fungal growth, decreased symptoms of gray mold disease and supressed fungal DCL transcripts. Also, Gu et al. (2019) determined that a β*_2_-tubulin* dsRNA derived from *Fusarium asiaticum* conferred plant resistance to multiple phytopathogens including *F. asiaticum*, *F. graminearum*, *F. tricinctum*, *F.* oxysporum, *F. fujikuroi, B. cinerea*, *M. oryzae*, and *Colletotrichum truncatum* and reduced resistance to carbendazim fungicide, inhibited fungal growth, and caused deformed and multiple-branching mycelium (Table 3). These studies indicate that SIGS is a broadly applicable tool for plant protection.

The SIGS method has recently been adopted against fungal wheat pathogens. For example, a spray application of a long non-coding dsRNA targeting the three fungal cytochrome *P450 lanosterol C-14*α*-demethylases* (*CYP51A*, *CYP51B*, *CYP51C*) inhibited the growth of *F. graminearum* in barley leaf tissue (Koch et al., 2016). Koch et al. (2020) sprayed whole barley plants with the 791-nt long *F. graminearum* CYP3-dsRNA. Hypocotyls of plants sprayed with CYP3-dsRNA developed fewer disease lesions compared to control plants, while CYP51-dsRNAs reduced *F. graminearum* infection areas by the factor of 80. In another study, Werner et al. (2020) sprayed dsRNAs targeting *ARGONAUTE* and *DICER* genes of *F. graminearum*, resulting in approximately 50% inhibition of fungal infection. In wheat, dsRNAs targeting the *Myo5* gene of phenamacril-resistant *F. asiaticum* were applied on plants and high levels of host resistance were observed (Song et al., 2018). Höfle et al. (2020) tested HIGS and SIGS approaches with dsRNA precursors of increasing length ranging from 400 to 1,500 nt to assess the effect of lengthy on gene silencing efficiency of *FgCYP51* genes. With HIGS-mediated disease control, they found no significant correlation between the length of the dsRNA precursor and the reduction of *F. graminearum* infection on *Arabidopsis*. Contrastingly, they observed that SIGS-mediated *F. graminearum* disease resistance negatively correlated with the length of the dsRNA construct that was sprayed, suggesting that increased size of the dsRNA interferes with uptake of dsRNAs by the fungus.

In soybean experiments, Hu et al. (2020) used both SIGS and HIGS to target genes of the rust pathogen *Phakopsora pachyrhizi*. First, eight genes involved in urediniospore germination or appressorium formation were targeted through a BPMV-based transient HIGS approach. HIGS against *ATC, GCS_H* and *RP_S16* reduced fungal biomass accumulation by 58–80%, respectively, and significantly reduced the development of asian soybean rust symptoms in soybean leaves (Table 3). In a SIGS study, spraying soybean leaves with dsRNA of *ATC, GCS_H*, and *RP_S16* achieved a reduction in the biomass of *P. pachyrhizi* of about 17.0, 20.9, and 25.1%, for each target gene, respectively (Hu et al., 2020). sRNA approaches could be particularly valuable for this pathogen, given the paucity of effective rust resistance genes in soybean germplasm Similarly, Nerva et al. (2020) applied dsRNA targeting *Botrytis cinerea (Bc)* genes *BcCYP51*, *Bcchs1*, and *BcEF2* by high pressure spraying of grapevine leaves and postharvest spraying of grape bunches. Their results showed a significant reduction in pathogen development only when *Bc* dsRNAs were applied.

## Host-induced gene silencing and spray-induced gene silencing against oomycetes

Compared to the extensive work on fungi, sRNA-based studies involving oomycetes are relatively scant. The first, by Sanju et al. (2015), targeted the *Avr3a* effector gene of the oomycete plant pathogen *Phytophthora infestans*. Disease severity decreased and production of siRNA molecules in host cells resulted in the inhibition of target gene expression in *P. infestans* through HIGS. Jahan et al. (2015) concluded that the choice of target genes and precursor hairpin-RNA is crucial for obtaining successful results from HIGS against the late blight pathogen *Phytophthora infestans*. The authors found that transgenic potato lines carrying targeted hairpin-RNA constructs to *Cellulose Synthase A2* (*PiCESA2*), *Pectinesterase* (*PiPEC*), and *Glyceraldehyde 3-phosphate dehydrogenase* (*PiGAPDH*) demonstrated weaker resistance to the late blight disease than transgenic potato lines expressing hairpin-RNA constructs targeting *P. infestans G protein*β*-subunit* (*PiGPB1*). HIGS studies with lettuce targeting the *Highly Abundant Message #34* (*HAM34*) or *Cellulose Synthase* (*CES1*) genes of the biotrophic downy mildew pathogen *Bremia lactucae* greatly reduced the growth and inhibited sporulation of the pathogen (Govindarajulu et al., 2015). Bilir et al. (2019) explored SIGS against an oomycete by targeting the *Cellulose synthase A3* (CesA3) gene of *Hyaloperonospora arabidopsidis* (*Hpa*), the downy mildew pathogen of *Arabidopsis*. They reported that antisense sRNAs targeting the *Hpa-CesA3* gene in Emoy2 and Cala2 isolates inhibited spore germination and thus infection of *Arabidopsis*, while sense sRNAs had no obvious effect on *Hpa* pathogenicity. When Haile et al. (2021) used dsRNA targeting the *DCL1 and DCL2* genes in grapevine downy mildew pathogen *Plasmopara viticola*, they observed reduction in the expression level of these genes as well as in the disease progress rate in the already established infection. Similar SIGS studies with dsRNA against the late blight pathogen *P. infestans* has been carried out by Kalyandurg et al. (2021). They have targeted several genes including *PiGPB1, PiHmp1, PiCut3*, and *PiEndo3* and through careful evaluation, they have concluded that some of these genes inhibited the pathogen development significantly. To improve the the efficacy of SIGS against *P. infestans*, Kalyandurg et al. (2021) recommended a few parameters including the use of adjuvants with dsRNA in the experiments.

The encouraging results from these studies should inspire more investment in sRNA-based approaches against oomycetes.

## sRNA-based protection of plants from nematodes

Host-induced gene silencing has successfully been used to control plant parasitic nematodes, including cyst nematodes (Alkharouf et al., 2007), root-knot nematodes (Huang et al., 2006), and recently, root lesion nematodes (Shivakumara et al., 2017; Chaudhary et al., 2019). For example, Huang et al. (2006) found that expression of dsRNAs in transgenic *Arabidopsis* that initiate siRNAs targeting the nematode parasitism gene *16D10* could reduce infestation by nematodes. Verma et al. (2018) demonstrated that the 30D08 effector protein is secreted from the nematode stylet into *Arabidopsis* cells and reported that plant-derived RNAi silencing of *30D08* decreased susceptibility to nematodes. Another study demonstrated that infection by root-knot nematodes is facilitated by the effector *Mi-msp2*. *Arabidopsis* lines expressing *Mi-msp2* dsRNA exhibited a significant reduction in nematodes (Joshi et al., 2019). Iqbal et al. (2020) used HIGS to study the effects of 20 genes involved in the RNA interference (RNAi) pathways in *Meloidogyne incognita*. Expression of *ego-1* and *mes-2* could not be eliminated, and expression of *xpo-1*, *pash-1*, *xpo-2*, *rha-1*, *ekl-4*, and *csr-1* was significantly elevated after RNAi treatment. However, there were significant decreases in expression of other genes indicating that genes can respond to RNAi differently, requiring an exhaustive assessment of target nematode genes by RNAi. Dinh et al. (2014) reported that HIGS-mediated downregulation of the putative effector gene *Mc16D10L* ensures a significant level of resistance against *M. chitwoodi* not only in *Arabidopsis* but also in stable transgenic lines of potato (Table 4).

**TABLE 4:** Summary of HIGS application for control of nematodes.

Pathogen	Host	RNA type	Target gene(s)	Main effect(s)	References
*M. incognita*, *M. javanica*, *M. arenaria*, *M. hapla*/Root-knot nematodes	Arabidopsis	dsRNA	*16D10*	Resistant to multiple root-knot nematode species	Huang et al., 2006
*Heterodera schachtii*/Sugar beet cyst nematode	Arabidopsis	dsRNA	*30D08*	Less susceptible to nematode infection	Verma et al., 2018
*M. hapla*, *M. arenaria* *M. graminicola*/Root-knot nematode	Arabidopsis	dsRNA	*Mi-msp2*	Reduced parasitism	Joshi et al., 2019
*Meloidogyne incognita*/Root-knot nematode	Arabidopsis	dsRNA	*rsd-3* *xpo-1* *xpo-2* *drh-3* *drsh-1* *pash-1* *vig-1* *ego-1* *smg-2* *smg-6* *eri-1* *gfl-1* *mut-7* *mes-2* *mes-6* *rha-1* *ekl-4* *ppw-2* *csr-1* *2242*	Significant reduction in nematode infectivity for drsh-1, mut-7, drh-3, rha-1, pash-1, and vig-1 genes	Iqbal et al., 2020
*Meloidogyne chitwoodi*/Root-knot nematode	Arabidopsis Potato	dsRNA	*16D10*	Enhanced resistance against *Meloidogyne chitwoodi* in two plants	Dinh et al., 2014
*Meloidogyne incognita*/Root-knot nematode	Chardonnay grape	siRNA	*16D10*	Inhibition of the nematode infection	Yang et al., 2013
*Meloidogyne incognita/*Southern root-knot nematode	Eggplant	dsRNA/ siRNA	*msp-18* *msp-20*	Improved resistance in eggplant	Shivakumara et al., 2017
*Meloidogyne incognita*/Root-knot nematode	Adzuki bean Tobacco	dsRNA	*Mi-sbp-1*	Slower nematode development and reduced parasitism on plants	Shivakumara et al., 2019
*Meloidogyne incognita/*Root-knot nematode	Eggplant	dsRNA/siRNA	*Mi-msp-1*	Enhanced nematode resistance	Chaudhary et al., 2019
*Heterodera glycines/*Soybean cyst nematode	Soybean	amiRNA	*J15* *J20* *J23*	Significant suppression on SCN cyst and egg populations	Tian et al., 2016
*Heterodera glycines/*Soybean cyst nematode	Soybean	hpRNA	*HgY25* *HgPrp17*	Significant reductions in numbers of SCN cysts and eggs	Tian et al., 2019
*Heterodera avenae*/Cereal cyst nematode	Wheat	dsRNA	*Galectin, cathepsin L, vap1, serpin, flp12, RanBPM chitinase*	Reduced nematode penetration, development and reproduction	Dutta et al., 2020
*Meloidogyne graminicola*/Root-knot nematode	Rice	hpRNA	*flp-1* *flp-12*	Reduction in gall formation, significant decrease in total number of endoparasites	Hada et al., 2020
*Meloidogyne* *incognita*/Root-knot nematode	Tobacco	dsRNA	*Chitin synthase, glucose-6-phosphate isomerase, trehalase*	Rreduced egg mass and egg number	Mani et al., 2020
*Meloidogyne incognita*/Root-knot nematode	*Arabidopsis*	hpRNA	*MiPDI1*	Reduced the amount of *M. incognita* infection	Zhao et al., 2020

hpRNA, hairpin RNA; amiRNA, artificial micro RNA; dsRNA, double-stranded RNA; siRNA, small interfering RNA.

A conserved root-knot nematode effector gene, *16D10*, was targeted by HIGS in transgenic grape hairy roots, showing successful inhibition of root-knot nematode infection (Yang et al., 2013). In addition, the HIGS suppression of *msp-18* and *msp-20* from *M. incognita* led to significant reduction in nematode multiplication (Shivakumara et al., 2017).

Tian et al. (2016) targeted three soybean cyst nematode genes (designated as *J15*, *J20*, and *J23*) by artificial microRNA (amiRNA), leading to downregulation of expression of these genes within soybean cyst nematode eggs in populations feeding on transgenic hairy roots. In addition, Tian et al. (2019) demonstrated that transgenic soybean lines expressing hairpin RNAi constructs targeting the *HgY25* and *HgPrp17* genes, related to reproduction and fitness of the nematodes, showed significant reductions (up to 73%) in eggs/g root in the T_3_ and T_4_ homozygous transgenic lines (Table 4). To date, SIGS has not been tested against nematodes.

## Current challenges of host-induced gene silencing and spray-induced gene silencing

From the extensive literature on HIGS, it is clear the transgenic approach using HIGS has been successful in revealing gene functions in a variety of plant pathogens and in protecting plants against diverse diseases. HIGS offers several potential advantages over other plant disease control methods. In principle, HIGS-based resistance does not require input from the grower, and thereby provides an effective crop protection strategy to replace costly and environmentally unfriendly chemical protection. HIGS-based resistance can be durable and long-lasting compared to conventional *R* genes that are often overcome quickly by compensatory mutations in pathogen *Avr* genes. Moreover, HIGS can be used to control multiple diseases of a given crop. Finally, new HIGS genes can be designed easily to keep pace with co-evolving pathogens.

Despite these advantages, commercially available crop plants with HIGS transgenes have not penetrated the marketplace. This may be due to the fact that HIGS requires transgene technology (Santala and Valkonen, 2018) and this is not accepted readily by the public in many countries, who are concerned about genetically modified organisms. For example, the literature indicates that European researchers are leaders in developing RNAi technology. However, Europe’s strict legislation against deployment of genetically engineered crops presents a stumbling block for the adoption of HIGS-based crop improvement. Moreover, genetic transformation protocols may not be accessible for some crop plants. As HIGS technology relies on the successful movement of siRNAs from the host plant to the pathogen, some pathogens may not be amenable to manipulation using this method. Off-target effects are another negative factor to be considered. Moreover, if the target region is not selected properly, the resultant functional redundancy and/or incomplete silencing of mRNA may result in failure. In addition, pathogens may cause diseases in only particular parts of the plants such as roots and fruits, and specific targeting of these tissues with HIGS system may not be possible. Recent studies indicate exosome-like extracellular vesicles are involved in sRNA trafficking between organisms (Cai et al., 2018). However, there are still gaps in our knowledge in this area and it is not known whether all plant-microbe interactions rely on the same mechanisms. Additional research into the fundamental mechanisms of trans-kingdom RNA silencing will undoubtedly improve efforts to streamline HIGS and SIGS as disease control tools.

Because SIGS technology has been used successfully on both monocots and dicots, it seems to be a potentially useful plant protection strategy. In comparison to current disease control methods, SIGS could be sustainable and environmentally friendly and it offers a rapid method suitable for both pre- and post-harvest plant protection. As a non-transgenic approach SIGS is potentially more acceptable to consumers. In addition, as SIGS technology targets specific genes using bioinformatic tools any off-target effects are reduced, and the sprays can be tailored toward particular pests or pathogens to enhance specificity.

However, as for HIGS, SIGS-based disease control also faces challenges: The effect of SIGS on plants may last for only a few days because of RNA degradation, and the level of protective RNA in the plant could be limited by uptake such that regular re-application of sRNA may be needed or limited by the size of dsRNA (Höfle et al., 2020) requiring a size optimization. Furthermore, it should be noted that efficiency of dsRNA uptake by the eukaryotic microbes and cell types could vary across fungal or oomycete species (Qiao et al., 2021). In addition, some of the delivery methods such as high pressure spraying of dsRNA may not yield the expected gene silencing (Uslu et al., 2020). The cost associated with manufacturing could be high. To overcome this, new approaches in this area, such as using bacteria to produce sRNAs, are being developed (Goodfellow et al., 2019); however, it is too early to judge whether this approach is commercially scalable. Also, although SIGS may work efficiently under the laboratory conditions, there have been very few large-scale field trials and only few delivery methods, including nanoclay, have been tested. Large scale production may rely on formulations containing synergists or co-formulants that stabilize dsRNA in the environment or enhance transport into the target cells. However, any active substance or product placed on the market to protect plants will be subject to authorization from the participating countries. This may cause a delay in the wide acceptance of SIGS technology as it may take some time for new regulatory frameworks to be developed. As SIGS technology is a new RNAi-based innovation, consumer acceptance has yet to be assessed.

## Conclusion and future prospects

In the near future, we should expect further developments in the commercial application of HIGS in regions where transgenic technology has been permitted. Similarly, an increasing number of studies have been carried out across the globe on a SIGS approach for disease control. However, several important aspects of SIGS, including risk assessment and legislation on the use of SIGS, need to be improved. As the genomic sequences of more plants and pathogens become increasingly available, designing HIGS and SIGS specific to the targets should get easier. Recent developments in clustered regularly interspaced short palindrome repeats (CRISPR)/CRISPR-associated protein 9 (Cas9) editing technology (Wada et al., 2020) may be combined with HIGS and SIGS to give more durable disease resistance in crop plants.

## Author contributions

ÖB, DG, YH, JM, and MT wrote the manuscript. All authors contributed to the article and approved the submitted version.
